# The association between obesity and back pain in nine countries: a cross-sectional study

**DOI:** 10.1186/s12889-015-1362-9

**Published:** 2015-02-11

**Authors:** Ai Koyanagi, Andrew Stickley, Noe Garin, Marta Miret, Jose Luis Ayuso-Mateos, Matilde Leonardi, Seppo Koskinen, Aleksander Galas, Josep Maria Haro

**Affiliations:** Research and Development Unit, Parc Sanitari Sant Joan de Déu, Fundació Sant Joan de Déu, Dr. Antoni Pujadas, 42, Sant Boi de Llobregat, Barcelona, Spain; Department of Human Ecology, Graduate School of Medicine, The University of Tokyo, Tokyo, Japan; Stockholm Centre on Health of Societies in Transition (SCOHOST), Södertörn University, Huddinge, Sweden; Department of Psychiatry, Universidad Autónoma de Madrid, Madrid, Spain; Instituto de Salud Carlos III, Centro de Investigación Biomédica en Red de Salud Mental, Cibersam, Spain; Department of Psychiatry, Hospital Universitario de La Princesa, Instituto de Investigación Sanitaria Princesa (IP), Madrid, Spain; Department of Neurology, Public Health and Disability Unit, Neurological Institute Carlo Besta IRCCS Foundation, Milan, Italy; National Institute for Health and Welfare, Helsinki, Finland; Department of Epidemiology, Jagiellonian University Medical College, Krakow, Poland

**Keywords:** Obesity, Back pain, Older population, Multi-country study, Developing country

## Abstract

**Background:**

The association between obesity and back pain has mainly been studied in high-income settings with inconclusive results, and data from older populations and developing countries are scarce. The aim of this study was to assess this association in nine countries in Asia, Africa, Europe, and Latin America among older adults using nationally-representative data.

**Methods:**

Data on 42116 individuals ≥50 years who participated in the Collaborative Research on Ageing in Europe (COURAGE) study conducted in Finland, Poland, and Spain in 2011–2012, and the World Health Organization’s Study on Global Ageing and Adult Health (SAGE) conducted in China, Ghana, India, Mexico, Russia, and South Africa in 2007–2010 were analysed. Information on measured height and weight available in the two datasets was used to calculate Body Mass Index (BMI). Self-reported back pain occurring in the past 30 days was the outcome. Multivariable logistic regression analysis was used to assess the association between BMI and back pain.

**Results:**

The prevalence of back pain ranged from 21.5% (China) to 57.5% (Poland). In the multivariable analysis, compared to BMI 18.5-24.9 kg/m^2^, significantly higher odds for back pain were observed for BMI ≥35 kg/m^2^ in Finland (OR 3.33), Russia (OR 2.20), Poland (OR 2.03), Spain (OR 1.56), and South Africa (OR 1.48); BMI 30.0-34.0 kg/m^2^ in Russia (OR 2.76), South Africa (OR 1.51), and Poland (OR 1.47); and BMI 25.0-29.9 kg/m^2^ in Russia (OR 1.51) and Poland (OR 1.40). No significant associations were found in the other countries.

**Conclusions:**

The strength of the association between obesity and back pain may vary by country. Future studies are needed to determine the factors contributing to differences in the associations observed.

## Background

The prevalence of back pain in the general population has been reported to be as high as 50% or more in both developed and developing countries [1,2]. Approximately 5-15% of back pain has a specific cause such as osteoporotic fracture, infection, or neoplasms, but the cause in the remainder of the cases is unknown [1]. According to the Global Burden of Disease 2010 study, lower back pain ranked first as the cause of global disability and sixth in terms of the overall disease burden [3]. In the context of global ageing, this is a major challenge as the prevalence and burden of low back pain increases with age [3].

The risk factors for back pain reported in previous studies include stress, anxiety, depression, heavy physical load [1], smoking [4], alcohol consumption [5], vitamin D deficiency [6], and obesity [7,8]. It has been postulated that obesity may cause back pain through mechanical load on the spine, systemic chronic inflammation [7], spine degeneration [9], or decreased blood flow to the spine due to atherosclerosis [10], while weight loss has been reported to lead to the resolution of back pain among the morbidly obese [11]. Although a recent meta-analysis demonstrated that overweight and obesity are associated with an increased risk for lower back pain [7], individual studies have been inconclusive [8].

The epidemiology of back pain may differ between settings as the type and prevalence of risk factors for back pain may vary. For example, heavy physical labour is more common in developing countries with entry into the workforce taking place at younger ages and physical labour being common even at older ages [12]. In addition, the association between obesity and back pain may differ as individuals in developing countries may have had a shorter period of exposure to obesity since the obesity epidemic generally started later in developing countries [13]. Because some of the adverse effects of obesity are known to become manifest as a result of cumulative exposure (e.g. arthritis) [14], some differences may be observed.

To date, studies on the association between obesity and back pain in developing countries are scarce, and there have been no multi-continent studies that have examined this association among older adults in countries at different stages of the socio-economic and nutritional transition using standardized data. This is an important research gap as the discrepant findings observed in previous studies may be due to the differences in the study design limiting comparability between studies. Also, whether regional differences exist in this association is unclear. In addition, despite rapid global ageing, there are very few studies on this topic among the older population. Thus, the aim of the current study was to assess the association between obesity and back pain among older adults using nationally-representative data from diverse settings.

## Methods

This study made use of data from the Collaborative Research on Ageing in Europe (COURAGE) and World Health Organization’s (WHO) Study on Global Ageing and Adult Health (SAGE) surveys. The COURAGE survey was conducted between 2011 and 2012 in Finland, Poland, and Spain, while the SAGE survey was undertaken in China, Ghana, India, Mexico, Russia, and South Africa between 2007 and 2010. The aim of these surveys was to create comparable databases with valid and reliable information on health and well-being in adult populations across the world. In particular, the SAGE countries broadly represent different geographical locations and levels of socio-economic and demographic transition. Using the World Bank classification at the time of the survey, these countries corresponded to high-, and middle-/low-income countries respectively [15]. Details of the survey methodology have been published elsewhere [16,17]. In brief, in order to obtain nationally-representative samples, a multistage clustered sampling design method was used. The sample consisted of adults aged ≥18 years with oversampling of those aged ≥50 years. Following a common research protocol across countries, trained interviewers conducted face-to-face interviews using a standard questionnaire to collect information on factors such as health status, quality of life, disability, and well-being. Quality control procedures were undertaken during the fieldwork [18]. The questionnaires were translated from English into the local languages, following the WHO translation guidelines for assessment instruments which consist of a forward translation, a targeted back-translation, review by a bilingual expert group, and detailed translation reports. All interviews in Mexico and the COURAGE survey countries were completed using a computer-assisted personal interview (CAPI), while a paper and pencil interview (PAPI) was used in the remaining countries with the exception of China where both CAPI and PAPI were used. Anthropometric data were also collected from respondents. A stadiometer and a routinely calibrated electronic weighting scale were used to measure height and weight respectively. If a respondent was unable to undertake the interview because of limited cognitive function, then a separate questionnaire was administered to a proxy respondent. The survey response rate ranged from 51% (Mexico) to 93% (China). Sampling weights were constructed to adjust for the population structure as reported by the United Nations Statistical Division and the National Institute of Statistics for the SAGE and COURAGE surveys respectively. Ethical approval for the SAGE and COURAGE surveys was obtained from the WHO Ethical Review Committee and local ethics research review boards (Helsinki and Uusimaa Hospital District, Finland; Jagiellonian University Medical College, Krakow, Poland; Parc Sanitari Sant Joan de Déu, Barcelona, Spain; La Princesa University Hospital, Madrid, Spain; Shanghai Municipal Centre for Disease Control and Prevention, Shanghai, China; Ghana Medical School, Accra, Ghana; International Institute of Population Sciences, Mumbai, India; National Institute of Public Health, Cuernavaca, Mexico; School of Preventive and Social Medicine, Russian Academy of Medical Sciences, Moscow, Russia; and Human Sciences Research Council, Pretoria, South Africa). The SAGE dataset is publically available online (http://www.who.int/healthinfo/sage/en/) and permission to use data from the COURAGE study was obtained from the country coordinators of this study in Finland, Poland, and Spain.

### Variables

Body mass index (BMI) was calculated as weight in kilograms divided by height in meters squared. Using the standard WHO definition, BMI was categorized as <18.5 kg/m^2^ (underweight), 18.5-24.9 kg/m^2^ (normal weight), 25.0-29.9 kg/m^2^ (overweight), 30.0-34.9 kg/m^2^ (obesity class I), and ≥35.0 kg/m^2^ (obesity class II+) [19]. Information on back pain was obtained by asking “Have you experienced back pain during the last 30 days?”. Those who answered ‘yes’ to this question were categorized as having back pain. Previous literature was used as a guide for the selection of variables that were used for adjustment. These included sex, age, completed education level (≤primary, secondary, ≥tertiary), wealth (assessed by quintiles based on country-specific income), past-12 months depression, physical activity, smoking status, and alcohol consumption [1,4,5,20]. DSM-IV algorithms for major depressive disorder were used to diagnose past-12 months depression. The Global Physical Activity Questionnaire was used to assess the level of physical activity using conventional cut-offs and categorized as low, moderate, and high (http://www.who.int/chp/steps/GPAQ/en/). For smoking, respondents were asked “Have you ever smoked tobacco or used smokeless tobacco?” and “Do you currently use (smoke, sniff or chew) any tobacco products such as cigarettes, cigars, pipes, chewing tobacco or snuff?” Those who answered ‘no’ to the first question were categorized as ‘never’ smokers, while those who answered ‘yes’ to both questions were regarded as ‘current’ smokers. Respondents who answered ‘yes’ to the first question but ‘no’ to the second were categorized as having ‘quit’. Alcohol consumption was assessed by the question “Have you ever consumed a drink that contains alcohol (such as beer, wine, spirits, etc.)?” Those who answered ‘no’ were categorized as ‘never’ drinkers. For those answering ‘yes’, a separate question asked about how many drinks of any alcohol beverage they had consumed on each day of the past week. Consumers of at least 4 (females) or 5 drinks (males) of any alcoholic beverage per day on at least one day in the past week were considered ‘heavy’ drinkers. Those who had ever consumed alcohol but were not heavy drinkers were categorized as ‘non-heavy’ drinkers [21].

### Statistical analysis

The analysis was restricted to those aged 50 years or older. Those respondents with proxy-provided information were excluded from the analysis due to the absence of some information pertaining to the current analysis. To account for the heterogeneity between countries, country-wise analyses were conducted. The crude and age-sex adjusted prevalence of back pain by country was calculated. Population pyramids from the United Nations for the year 2010 (http://esa.un.org/wpp/Excel-Data/population.htm) were used as the standard population to estimate the age- and sex-adjusted prevalence of back pain. Furthermore, the crude prevalence of demographic, lifestyle factors, and depression was calculated by the presence or absence of back pain. Multivariable logistic regression analysis was used to assess the association between BMI (independent variable) and back pain (dependent variable). As the aim of the current study was to compare normal and higher categories of BMI in terms of their association with back pain, respondents with a BMI < 18.5 kg/m^2^ (underweight) were excluded from the regression analyses. This resulted in 4.3% (China), 0.5% (Finland), 15.2% (Ghana), 38.8% (India), 0.6% (Mexico), 1.1% (Poland), 1.1% (Russia), 3.1% (South Africa), and 0.6% (Spain) of the subjects being excluded. The model adjusted for sex, age, education, wealth, depression, physical activity, smoking status, and alcohol consumption. Pooled estimates were also calculated but only for the COURAGE survey counties because geographical location and income levels were similar in these countries and so was the association between obesity and back pain. The pooled estimates were adjusted for country by including countries as dummy variables. No attempt was made to obtain pooled estimates for the SAGE survey countries as their income levels and geographical locations were heterogeneous and the association between obesity and back pain was not similar between countries. In order to generate nationally-representative estimates, in all analyses, the sample weighting and the complex study design were taken into account with Taylor linearization methods. The analyses were performed with Stata version 12.1 (Stata Corp LP, College Station, Texas). The level of statistical significance was set at P < 0.05.

## Results

**Figure 1:**
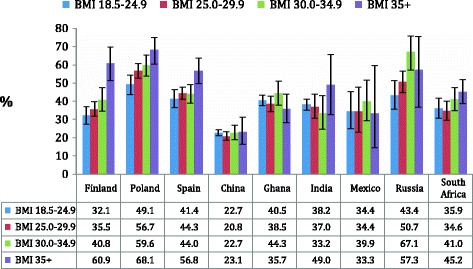
**Crude prevalence of back pain by body mass index.** Abbreviation: BMI Body mass index. Prevalence based on weighted sample. Bars denote 95% confidence intervals.

After the exclusion of those under age 50 years, the total sample size was 42116 [China (13175), Finland (1452), Ghana (4305), India (6560), Mexico (2313), Poland (2910), Russia (3938), South Africa (3838), and Spain (3625)]. The prevalence of back pain is illustrated in Table 1. There was little difference between the crude and age-sex adjusted estimates. The crude prevalence ranged from 21.5% (China) to 57.5% (Poland). The crude prevalence of back pain by BMI category is shown in Figure 1. A dose dependent-like increase in back pain associated with higher BMI was observed in Finland, Poland, and Spain. The characteristics of the study sample by the presence of back pain are presented in Table 2. A particularly high prevalence of BMI ≥ 35 kg/m^2^ was observed among those with back pain as compared to those without back pain in Finland (13.0% vs. 5.0%), Poland (11.7% vs. 7.1%), and Spain (10.4% vs. 6.4%). The association between BMI, depression, demographic or lifestyle factors, and back pain is shown in Table 3. Compared to normal weight (BMI 18.5-24.9 kg/m^2^), significantly higher odds for back pain were observed for the following BMI categories: overweight (BMI 25.0-29.9 kg/m^2^) in Poland (OR 1.40) and Russia (OR 1.51); obesity class I (BMI 30.0-34.9 kg/m^2^) in Poland (OR 1.47), Russia (OR 2.76), and South Africa (OR 1.51); obesity class II+ (BMI ≥35 kg/m^2^) in Finland (OR 3.33), Poland (OR 2.03), Spain (OR 1.56), Russia (OR 2.20), and South Africa (OR 1.48). The pooled ORs for the countries in the COURAGE survey were: overweight [OR 1.30 (95%CI 1.10-1.54); p < 0.002], obesity class I [OR 1.33 (95%CI 1.09-1.61); p = 0.005], and obesity class II+ [OR 1.95 (95%CI 1.49-2.56); p < 0.001].

**Table 1:** **Prevalence of back pain among adults aged 50 years or over**

**Survey**	**Country**	**Crude**		**Age-sex adjusted**	
COURAGE	Finland	38.0	(35.8-40.4)	37.3	(35.2-39.5)
	Poland	57.5	(54.8-60.1)	56.3	(53.5-59.1)
	Spain	45.1	(42.2-48.0)	43.5	(40.3-46.7)
SAGE	China	21.5	(19.9-23.2)	21.5	(19.9-23.3)
	Ghana	40.5	(38.2-42.8)	40.1	(37.9-42.4)
	India	39.2	(36.5-42.0)	39.8	(37.0-42.7)
	Mexico	35.5	(28.9-42.8)	35.8	(30.0-42.1)
	Russia	53.6	(48.6-58.5)	52.3	(47.5-57.0)
	South Africa	39.3	(36.0-42.7)	39.1	(35.9-42.4)

*Abbreviations*: *COURAGE* Collaborative Research on Ageing in Europe, *SAGE* WHO Study on Global Ageing and Adult Health.

Data are % (95% confidence intervals). Prevalence based on weighted sample.

**Table 2:** **Characteristics of the study sample by presence of back pain**

	**COURAGE survey**						**SAGE survey**											
	**Finland**		**Poland**		**Spain**		**China**		**Ghana**		**India**		**Mexico**		**Russia**		**S. Africa**	
**Back pain**	**No**	**Yes**	**No**	**Yes**	**No**	**Yes**	**No**	**Yes**	**No**	**Yes**	**No**	**Yes**	**No**	**Yes**	**No**	**Yes**	**No**	**Yes**
BMI (kg/m^2^)																		
<18.5	0.6	0.2	1.4	0.9	0.5	0.6	4.4	3.9	15.1	15.3	37.7	40.5	0.6	0.5	1.5	0.8	3.1	3.4
18.5-24.9	33.9	26.8	28.2	20.8	23.7	20.7	60.0	62.3	54.8	55.6	48.8	47.0	21.4	20.4	28.8	19.3	24.6	21.4
25.0-29.9	42.5	39.2	39.4	39.6	45.3	44.8	29.8	27.7	20.3	18.9	10.9	10.0	50.4	48.1	43.1	38.7	28.4	23.3
30.0-34.9	18.0	20.8	23.9	27.0	24.0	23.4	4.9	5.1	5.9	7.0	1.8	1.4	19.7	23.8	16.1	28.6	22.1	23.8
≥35.0	5.0	13.0	7.1	11.7	6.4	10.4	0.9	1.0	3.9	3.3	0.7	1.1	7.9	7.2	10.7	12.5	21.9	28.1
Age (years)																		
50-59	34.5	31.3	43.5	34.4	34.8	29.0	46.4	41.2	42.0	36.6	51.3	44.4	52.3	43.3	53.7	37.8	52.9	44.7
60-69	35.8	35.2	31.4	34.1	28.9	27.4	31.1	34.6	27.5	27.6	30.2	32.0	23.2	30.4	23.6	25.4	29.3	33.4
70-79	19.3	21.6	17.7	21.6	27.2	32.0	18.3	19.7	21.1	25.8	14.1	18.8	17.5	18.5	15.8	26.9	13.3	14.8
≥80	10.5	11.9	7.4	9.9	9.2	11.6	4.2	4.5	9.4	10.1	4.3	4.8	7.0	7.7	6.9	9.9	4.5	7.1
Female	49.7	60.4	52.5	59.5	43.4	66.1	48.1	58.3	45.0	51.7	41.9	60.0	49.9	59.7	58.9	62.7	52.7	61.4
Education																		
≥Tertiary	27.5	23.4	19.0	13.3	13.4	7.6	5.1	2.3	4.1	2.9	7.0	2.2	9.0	6.5	22.8	14.4	7.1	3.7
Secondary	56.1	57.9	60.4	58.4	28.7	21.9	34.4	26.1	22.8	18.7	21.9	14.0	9.9	16.6	72.1	76.1	24.5	20.0
≤Primary	16.4	18.7	20.6	28.3	57.9	70.5	60.5	71.6	73.1	78.4	71.1	83.8	81.1	76.9	5.1	9.5	68.3	76.3
Wealth																		
Poorest	22.2	24.5	20.9	27.8	22.0	22.7	14.8	22.2	20.2	15.5	16.1	21.4	11.6	21.7	16.3	16.0	20.7	19.3
Poorer	25.6	29.8	21.4	18.6	21.4	26.8	17.3	21.4	18.5	20.0	19.7	19.2	21.4	14.9	21.4	19.3	21.4	21.2
Middle	18.6	20.8	17.6	18.9	19.2	21.5	19.9	21.6	17.8	24.5	18.3	19.6	18.0	13.9	17.6	20.5	18.7	18.5
Richer	18.3	14.8	26.8	19.2	19.9	17.6	24.3	19.4	20.6	20.6	18.6	21.3	17.4	15.5	22.9	18.8	19.3	21.0
Richest	15.3	10.1	19.6	15.5	17.6	11.5	23.7	15.4	22.9	19.3	27.4	18.5	22.5	34.1	23.3	25.4	22.1	20.0
Depression	10.5	23.4	5.9	19.2	18.0	36.6	0.9	2.9	2.8	15.6	11.9	22.3	14.1	21.9	2.3	8.5	2.7	8.0
Physical activity																		
High	43.2	39.6	47.4	49.5	32.3	28.5	43.7	47.0	59.9	64.6	52.8	51.4	40.5	39.1	57.0	58.3	30.0	25.7
Moderate	33.5	28.6	19.3	19.9	41.5	33.3	27.7	26.9	13.5	11.1	23.9	21.0	22.1	23.0	15.0	16.5	11.8	13.2
Low	23.3	31.8	33.3	30.6	26.2	38.2	28.6	26.0	26.7	24.3	23.3	27.6	37.4	37.9	28.0	25.2	58.2	61.1
Smoking status																		
Never	35.4	35.5	45.7	43.8	47.9	60.0	63.6	65.6	75.2	75.1	44.5	46.5	63.1	56.2	71.8	67.7	66.6	67.1
Current	17.0	17.6	27.2	25.2	22.0	17.2	29.8	27.8	10.9	10.4	51.1	48.4	18.4	23.7	21.0	21.7	22.9	25.3
Quit	47.6	46.9	27.1	30.9	30.2	22.9	6.6	6.5	13.9	14.5	4.4	5.1	18.5	20.1	7.2	10.6	10.6	7.6
Alcohol consumption																		
Never	8.8	11.5	15.4	14.7	27.6	35.6	67.5	67.3	42.1	45.2	84.5	86.1	48.9	48.9	32.9	25.7	76.8	76.1
Non-heavy	77.4	74.8	79.1	77.9	69.5	62.7	25.4	26.0	55.4	53.1	14.9	13.3	46.4	46.1	62.5	68.6	20.2	19.2
Heavy	13.8	13.7	5.5	7.4	2.9	1.7	7.1	6.6	2.5	1.6	0.6	0.6	4.7	5.0	4.6	5.7	3.1	4.7

*Abbreviations*: *COURAGE* Collaborative Research on Ageing in Europe, *SAGE* WHO Study on Global Ageing and Adult Health, *S. Africa* South Africa, *BMI* Body Mass Index.

Data are %. Prevalence based on weighted sample.

**Table 3:** **Association between body mass index or other factors, and back pain among adults aged 50 years or over estimated by multivariable logistic regression**

			**COURAGE survey**			**SAGE survey**					
**Characteristic**	**Reference**		**Finland**	**Poland**	**Spain**	**China**	**Ghana**	**India**	**Mexico**	**Russia**	**S. Africa**
BMI (kg/m^2^)	18.5-24.9	25.0-29.9	1.19	1.40*	1.23	0.96	0.90	1.01	1.54	1.51*	1.08
			(0.85-1.67)	(1.07-1.83)	(0.95-1.59)	(0.85-1.08)	(0.72-1.13)	(0.76-1.34)	(0.86-2.75)	(1.05-2.16)	(0.81-1.44)
		30.0-34.9	1.39	1.47*	1.13	1.08	1.23	0.96	1.41	2.76***	1.51*
			(0.93-2.06)	(1.10-1.96)	(0.85-1.51)	(0.87-1.34)	(0.92-1.64)	(0.61-1.51)	(0.71-2.78)	(1.83-4.17)	(1.04-2.20)
		≥35.0	3.33***	2.03***	1.56*	1.00	0.90	1.21	1.17	2.20*	1.48*
			(2.09-5.30)	(1.34-3.09)	(1.03-2.35)	(0.65-1.55)	(0.59-1.36)	(0.56-2.62)	(0.54-2.57)	(1.15-4.21)	(1.02-2.16)
Age (years)	50-59	60-69	0.97	1.40**	1.15	1.17**	1.14	1.20	1.21	1.81**	1.35*
			(0.69-1.36)	(1.10-1.78)	(0.88-1.51)	(1.05-1.31)	(0.95-1.36)	(0.95-1.52)	(0.73-2.00)	(1.23-2.67)	(1.04-1.75)
		70-79	1.24	1.66**	1.13	1.06	1.29*	1.41*	1.25	3.11***	1.57*
			(0.79-1.94)	(1.14-2.40)	(0.87-1.46)	(0.92-1.22)	(1.01-1.66)	(1.01-1.96)	(0.74-2.11)	(1.99-4.86)	(1.10-2.24)
		≥80	1.16	1.75**	1.18	0.95	1.05	1.43	1.30	3.53***	1.56
			(0.68-2.00)	(1.20-2.55)	(0.81-1.70)	(0.67-1.34)	(0.75-1.47)	(0.91-2.25)	(0.62-2.74)	(1.88-6.64)	(0.89-2.74)
Sex	Male	Female	1.53**	1.45**	2.54***	1.84***	1.32**	2.00***	2.80***	1.40	1.37*
			(1.17-1.99)	(1.14-1.84)	(2.05-3.15)	(1.58-2.15)	(1.10-1.59)	(1.56-2.58)	(1.59-4.91)	(0.93-2.10)	(1.02-1.86)
Education	≥Tertiary	Secondary	1.03	1.35	0.98	1.40	0.97	1.29	1.67	1.47*	1.52
			(0.78-1.34)	(0.98-1.85)	(0.68-1.40)	(0.96-2.04)	(0.58-1.63)	(0.80-2.08)	(0.59-4.71)	(1.04-2.08)	(0.79-2.90)
		≤Primary	0.96	1.50*	1.32	1.72*	1.09	1.79*	1.31	1.67	2.01*
			(0.66-1.38)	(1.02-2.20)	(0.89-1.98)	(1.10-2.68)	(0.66-1.82)	(1.14-2.83)	(0.51-3.34)	(0.97-2.85)	(1.04-3.90)
Wealth	Middle	Poorest	0.75	1.06	0.83	1.23	0.55***	1.01	2.26*	0.83	0.97
			(0.49-1.14)	(0.76-1.49)	(0.61-1.12)	(0.97-1.57)	(0.41-0.74)	(0.70-1.47)	(1.17-4.35)	(0.52-1.33)	(0.66-1.42)
		Poorer	0.98	1.11	0.92	1.07	0.71*	0.79	0.59	0.79	1.20
			(0.66-1.44)	(0.76-1.62)	(0.72-1.18)	(0.86-1.33)	(0.54-0.94)	(0.55-1.13)	(0.30-1.13)	(0.42-1.48)	(0.78-1.85)
		Richer	0.79	0.73	0.88	0.76*	0.76*	1.18	0.93	0.82	0.93
			(0.54-1.15)	(0.52-1.03)	(0.61-1.26)	(0.58-1.00)	(0.58-0.99)	(0.80-1.76)	(0.49-1.75)	(0.43-1.56)	(0.64-1.37)
		Richest	0.65	0.85	0.77	0.68**	0.64***	0.74	1.73	1.01	1.03
			(0.40-1.05)	(0.60-1.22)	(0.57-1.04)	(0.52-0.89)	(0.50-0.83)	(0.53-1.03)	(0.82-3.68)	(0.58-1.75)	(0.64-1.66)
Depression	No	Yes	2.51*	5.23***	2.51***	3.08***	6.74***	2.40***	2.09*	7.66***	3.89***
			(1.26-5.02)	(3.05-8.98)	(1.75-3.58)	(2.07-4.57)	(4.33-10.47)	(1.82-3.16)	(1.07-4.10)	(2.47-23.73)	(1.84-8.22)
Physical activity	High	Moderate	0.81	0.96	0.85	0.99	0.78	0.80	0.90	1.06	1.69*
			(0.61-1.07)	(0.72-1.28)	(0.67-1.07)	(0.85-1.16)	(0.57-1.06)	(0.64-1.00)	(0.50-1.64)	(0.72-1.55)	(1.10-2.61)
		Low	1.21	0.82	1.33*	0.89	0.83	1.05	0.74	0.72	1.33
			(0.86-1.69)	(0.62-1.09)	(1.02-1.73)	(0.73-1.09)	(0.64-1.07)	(0.79-1.39)	(0.44-1.25)	(0.48-1.07)	(0.94-1.88)
Smoking status	Never	Smoker	1.46	1.12	1.11	1.28**	0.94	1.18	1.93	1.30	1.14
			(0.97-2.21)	(0.83-1.50)	(0.86-1.43)	(1.08-1.52)	(0.70-1.27)	(0.93-1.50)	(0.98-3.82)	(0.73-2.33)	(0.80-1.64)
		Quit	1.34*	1.30	1.19	1.32*	1.25	1.33	1.58	1.79	0.84
			(1.01-1.77)	(0.98-1.72)	(0.85-1.65)	(1.02-1.71)	(0.95-1.64)	(0.76-2.35)	(0.82-3.05)	(0.95-3.36)	(0.52-1.34)
Alcohol	Never	Non-heavy	0.76	1.16	1.05	1.21*	0.90	1.25	1.39	1.85**	1.17
consumption			(0.47-1.22)	(0.86-1.57)	(0.74-1.47)	(1.00-1.45)	(0.75-1.09)	(0.92-1.69)	(0.81-2.39)	(1.23-2.77)	(0.85-1.62)
		Heavy	0.85	1.90*	0.99	1.10	0.58	0.85	1.38	3.08**	1.69
			(0.46-1.58)	(1.09-3.30)	(0.40-2.47)	(0.83-1.45)	(0.32-1.03)	(0.24-3.04)	(0.40-4.80)	(1.45-6.56)	(0.90-3.17)

*Abbreviations*: *COURAGE* Collaborative Research on Ageing in Europe, *SAGE* WHO Study on Global Ageing and Adult Health, *S. Africa* South Africa, *BMI* Body Mass Index.

Data are adjusted OR (95% confidence intervals). Models are adjusted for all covariates in the table.

*p < 0.05, **p < 0.01, ***p < 0.001.

Older age was clearly associated with back pain in Poland and Russia but in other countries, either no significant association was found or only some age categories had significant results as compared to the youngest age group. Females were significantly more likely to complain of back pain in all countries except Russia. Lower education was significantly associated with back pain in Poland, China, India, and South Africa. A higher level of wealth was significantly protective against back pain in China, Ghana, and Mexico although the association observed in Ghana was U-shaped where the poorer also had lower odds for back pain as compared to those in the middle wealth quintile. Depression was significantly associated with back pain in all countries with ORs ranging from 2.09 (Mexico) to 7.66 (Russia). Compared to those engaging in high levels of physical activity, those with low and moderate levels of physical activity were 1.33 and 1.69 times significantly more likely to have back pain in Spain and South Africa respectively. As compared to never smoking, current smoking was significantly associated with back pain only in China (OR 1.28), while past smoking was significantly associated with back pain in Finland (OR 1.34) and China (OR 1.32). Finally, alcohol consumption was significantly associated with back pain in Poland, China, and Russia.

## Discussion

To the best of our knowledge, this is the first multi-continent study to examine the association between BMI and back pain among older adults. Our study shows that the association between BMI and back pain may differ by context. Significant associations between high BMI and back pain were only observed in Finland, Poland, Spain, Russia, and South Africa but no significant associations were observed in the other countries. The strength of this study is the use of large nationally-representative data obtained by standardized questionnaires and measured BMI across a variety of settings.

Several limitations should be borne in mind however, when interpreting the results. The data in this study, with the exception of BMI, were self-reported. Thus, reporting bias could have affected our results. The reporting of back pain, for example, is conditioned by individual perceptions that can be affected by specific cultural and environmental conditions. In particular, the differences in the prevalence of back pain across countries might stem from methodological, linguistic and cultural variability in the understanding and definition of back pain across country settings [22]. For example, if pain is as claimed, a “culturally defined physiological and psychological experience” [23], then it is possible that the term ‘pain’ might itself have been understood differently [24] by respondents in different countries leading to different interpretations and responses to our study question. Indeed, this cultural variability might even stretch to differences in what exactly constitutes the ‘back’. An earlier study which looked at back pain in Germany and Britain for example, highlighted that in the former there was a different conception of what constitutes the ‘back’ with no equivalent to the concept of the ‘low back’ found in Britain [25]. Regarding the methodology, our question asked about the occurrence of back pain “During the last 30 days” without attempting to determine how long the pain lasted, whether it was chronic or acute, or how it impacted on the daily activity of the subject. Again, this might have been problematic as a previous review has shown for example, that different definitions of the duration of back pain (i.e. on the day of a survey vs. more than two weeks duration) can lead to a large difference in the reported prevalence of pain [26]. Thus, taken together, this body of research not only highlights that a number of factors might affect the reporting of pain in different locations, but it also suggests that future cross-country research should be cognizant of the potential for cultural differences in the interpretation and reporting of pain, and how more precise definitions of pain, where it is occurring, its duration and intensity may lead to better comparative prevalence estimates across countries. In addition, most previous research on obesity and back pain has focused on lower back pain. Thus, the results of our study may not be directly comparable with those from other studies as we focused on back pain with no specification of location. Moreover, we did not have any information on stress, anxiety, percent body fat, and vitamin D deficiency which have been associated with both obesity and back pain in previous studies [1,6,20,27-29]. Thus, their independent and potentially confounding effects remain unknown. Finally, as with all cross-sectional research, it is impossible to establish causality. For example, back pain may be the cause of obesity as a result of less physical activity.

The prevalence of back pain increased with age in Poland and Russia but no clear patterns were observed in the other countries. Previous studies have reported an increasing trend with age or a decrease after age 65 years [3,30]. The different patterns observed between countries may be related to differences in the frequency of factors such as increased tolerance or decreased perception of pain associated with ageing, or activities which cause back pain [30]. The finding that female sex, lower wealth, and education were associated with back pain in most or some countries has been previously reported [1]. Lower socioeconomic status may be associated with back pain through heavy manual work or less access to health facilities. The reason for the U-shaped association in Ghana where both low and high socioeconomic status were associated with lower odds for back pain is unclear and is an area for future research.

In our study, depression, which has also been associated with back pain in previous studies [20], was the strongest and most consistent correlate of back pain across countries suggesting that mental health may be an important determinant of back pain in the countries studied. Compared to high levels of physical activity, low and moderate physical activity were associated with significantly higher odds for back pain in Spain and South Africa respectively. Inconsistent results have also been found in previous studies and the association seems to depend on the type of physical activity [31]. Furthermore, although not statistically significant, a U-shaped association between physical activity and back pain was observed notably in countries such as Finland and Spain where the moderate category had the lowest odds for back pain. This U-shaped association where both low and high levels of exercise are associated with a higher risk of back pain as compared with a moderate amount of exercise has been reported previously [32,33]. The exact mechanism underlying the association between low physical activity and higher odds for back pain is uncertain but at least one explanation may be that those with back pain are avoiding physical activity due to fear that it would exacerbate the pain [34].

Current smoking was only associated with back pain in China, and past smoking was associated with back pain in Finland and China. Similar findings have been previously reported [4]. Heavy alcohol consumption was associated with back pain in Poland and Russia, while non-heavy alcohol consumption was associated with back pain in China and Russia. The regional variations observed in the association between lifestyle factors and back pain may be due to factors such as differences in the type of physical activity undertaken, quantity of cigarettes smoked, or the amount and quality of alcohol consumed. Alternatively, as smoking and alcohol consumption have been identified as coping mechanisms for stress, and stress, in turn, is associated with back pain, this may be a reflection of different types of stress coping strategy used in different populations [35].

It is possible that the inclusion of different control variables in the model (such as the ‘lifestyle’ variables discussed above) might have affected the association between BMI and pain in different ways. To assess this, we also conducted an exploratory hierarchical analysis that examined the effect of including different covariates in the model sequentially by comparing the BMI odds ratios in the univariable and subsequent models. However, adjustment for other factors associated with back pain did not appreciably affect the BMI odds ratios across the different models (data not shown).

A review article, which included 56 studies conducted between 1965 and 1997, concluded that there was insufficient evidence to assess whether obesity causes low back pain [8]. In a recent meta-analysis published in 2010, which included 33 studies, obesity was associated with 1.33 (cross-sectional studies) and 1.53 (cohort studies) times higher odds for lower back pain [7]. A recent large population-based 11-year follow-up study conducted in Norway found that among those without low back pain at baseline, the risk for developing low back pain was 1.34 and 1.22 times significantly higher for men and women when BMI ≥ 30 kg/m^2^ was compared to BMI < 25 kg/m^2^ [36]. It has been suggested that obesity might cause back pain through a number of different mechanisms including decreased blood flow to the spine through the effects of atherosclerosis [10], spine degeneration [9], or as a result of mechanical stress, and changes in metabolism, or via inflammatory pathways [7].

In our study, significant associations between overweight and/or obesity and back pain were observed in Finland, Poland, Spain, Russia, and South Africa but not in the other countries. The reason why obesity was associated with back pain only in some countries but not in others is unclear but several reasons may be speculated. First of all, since the obesity epidemic started earlier in some settings [13], some countries may have had an average younger age of obesity onset resulting in a longer cumulative exposure to obesity. Alternatively, this regional difference may at least partly be a function of survival. The obese in low or middle-income countries may not live long, while those in high-income countries may live longer due to the better care of cardiovascular diseases for which the obese are at higher risk. Longer exposure to obesity may lead to a higher risk of back pain through its cumulative mechanical load on the spine. Next, this association may be related to the link between stress and back pain. Stress or anxiety may be common underlying factors for obesity and back pain where the link between stress and obesity might be through the effects of overeating [37,38]. Specifically, overeating, which is sometimes used as a coping strategy to mitigate stress or anxiety, may be more commonly used in some contexts, while some societies tend to have higher levels of stress [39]. Also, fat mass but not lean mass has been associated with low back pain [29]. The difference in average percent body fat for a given BMI category between ethnic groups may also have contributed to some of the differences observed. However, Asians generally have higher fat levels compared to Caucasians for the same BMI [40], and thus, our study does not necessarily support this hypothesis as no significant association between obesity and back pain was observed in the Asian countries. Finally, vitamin D deficiency has been associated with low back pain [6]. It may be that obesity is associated with different levels of vitamin D deficiency depending on the context [41].

## Conclusion

Overweight and/or obesity were associated with significantly higher odds for back pain among those aged 50 and above in Finland, Poland, Spain, Russia, and South Africa. Our study indicates that the association between obesity and back pain may be setting-dependent. This suggests that weight loss interventions to prevent back pain may have limited effects in some settings. Further research is now needed to more precisely determine the underlying factors that are contributing to the differences observed between countries. In particular, studies may need to take into account factors that were not measured in our study or in most previous studies such as anxiety, stress-related eating, body fat mass, and the level of vitamin D deficiency.

## Abbreviations

BMI: Body mass index
COURAGE: Collaborative research on ageing in Europe
SAGE: WHO Study on Global Ageing and Adult Health
